# Phagocytic microglia and macrophages in brain injury and repair

**DOI:** 10.1111/cns.13899

**Published:** 2022-06-25

**Authors:** Fang Yu, Yangfan Wang, Anne R. Stetler, Rehana K. Leak, Xiaoming Hu, Jun Chen

**Affiliations:** ^1^ Geriatric Research, Education and Clinical Center Veterans Affairs Pittsburgh Health Care System Pittsburgh Pennsylvania USA; ^2^ Pittsburgh Institute of Brain Disorders & Recovery and Department of Neurology University of Pittsburgh Pittsburgh Pennsylvania USA; ^3^ Graduate School of Pharmaceutical Sciences School of Pharmacy, Duquesne University Pittsburgh Pennsylvania USA

**Keywords:** acute brain injury, brain repair, microglia/macrophage, phagocytosis

## Abstract

**Aims:**

Phagocytosis is the cellular digestion of extracellular particles, such as pathogens and dying cells, and is a key element in the evolution of central nervous system (CNS) disorders. Microglia and macrophages are the professional phagocytes of the CNS. By clearing toxic cellular debris and reshaping the extracellular matrix, microglia/macrophages help pilot the brain repair and functional recovery process. However, CNS resident and invading immune cells can also magnify tissue damage by igniting runaway inflammation and phagocytosing stressed—but viable—neurons.

**Discussion:**

Microglia/macrophages help mediate intercellular communication and react quickly to the “find‐me” signals expressed by dead/dying neurons. The activated microglia/macrophages then migrate to the injury site to initiate the phagocytic process upon encountering “eat‐me” signals on the surfaces of endangered cells. Thus, healthy cells attempt to avoid inappropriate engulfment by expressing “do not‐eat‐me” signals. Microglia/macrophages also have the capacity to phagocytose immune cells that invade the injured brain (e.g., neutrophils) and to regulate their pro‐inflammatory properties. During brain recovery, microglia/macrophages engulf myelin debris, initiate synaptogenesis and neurogenesis, and sculpt a favorable extracellular matrix to support network rewiring, among other favorable roles. Here, we review the multilayered nature of phagocytotic microglia/macrophages, including the molecular and cellular mechanisms that govern microglia/macrophage‐induced phagocytosis in acute brain injury, and discuss strategies that tap into the therapeutic potential of this engulfment process.

**Conclusion:**

Identification of biological targets that can temper neuroinflammation after brain injury without hindering the essential phagocytic functions of microglia/macrophages will expedite better medical management of the stroke recovery stage.

## Graphical abstract

Find‐me, Eat‐me, and Don’t‐eat‐me signals implicated in microglia/macrophage phagocytosis pathways after brain injury. Find‐me signals are instrumental in the recognition of chemotactic modulators, such as nucleotides, CX3CL1, and other molecular signals, including S1P/LPC and HMGB1 released by dying/dead brain cells. Find‐me signals are recognized by microglia/macrophage receptors P2Y12, TLR, CX3CR1 and S1PR, resulting in chemotaxis of microglia/macrophages to injured brain areas. Eat‐me signals are released or expressed by dead/dying brain cells (mostly neurons). When phosphatidylserine (PS) is flipped and exposed on the outer layer of the cell membrane, it is recognized by multiple microglia/macrophage receptors that initiate the phagocytosis process. Don’t‐eat‐me signal pathways inhibit phagocytosis and involve sialylated glycoproteins and lipids. CD47 interacts with microglial receptor signal‐regulatory protein alpha (SIRPα) to inhibit phagocytosis.

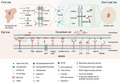

## 1 INTRODUCTION

Phagocytosis is an essential biological process involving the engulfment of extracellular particles, such as bacteria or cell debris, into the phagocytic cell, where the enveloped particles undergo lysosomal degradation.^1^ Microglia/macrophages are the professional phagocytic cell populations of the central nervous system (CNS), with critical roles in shaping neurodevelopment, maintaining homeostasis, and regulating pathological processes such as acute brain injuries and other neurological disorders.

Acute brain injuries are associated with a high risk of mortality and disability.^2^ In addition to the initial site of injury, waves of cell dysfunction/death ripple through surrounding tissues and interconnected circuits. The buildup of toxic cell debris initiates profound inflammatory responses within the brain and sends distress signals to the body to recruit peripheral immune cells to the site of injury, including neutrophils and blood‐derived monocytes/macrophages.^3^, ^4^, ^5^ A persistent and exaggerated immune response triggered by dead brain cells and cellular debris may trigger self‐amplifying, uncontrolled neuroinflammatory cascades and hinder functional recovery after stroke.^6^ On the contrary, effective clearance of dead cells, myelin debris, and harmful cell components mitigates the release of cytotoxic and pro‐inflammatory markers, a process that is essential for functional recovery following brain injuries.^7^, ^8^ Although peripheral immune cell infiltration is a major contributor in the pathogenesis of secondary brain injury,^9^ microglia are capable of engulfing infiltrating immune cells and their cellular components^10^ to control the accumulation of peripheral infiltrating immune cells in the injured brain.^4^

Microglia/macrophage phagocytosis is the backbone of a natural compensatory response to brain injuries.^6^, ^11^, ^12^ Initially, phagocytosis was thought to be only beneficial but mounting evidence suggests that aspects of poststroke phagocytosis might also be detrimental.^7^, ^13^, ^14^, ^15^ For example, peri‐infarct stressed brain cells, including neurons that have the potential to recover and live, can also be phagocytosed by microglia/macrophages, thereby exacerbating neuronal loss and contributing to delayed brain atrophy and neurodegenerative changes.^16^ This detrimental aspect of phagocytosis following brain injuries might be further amplified by aging, when overactivated phagocytosis is associated with cognitive and memory impairments and progressive brain atrophy.^4^, ^17^ The existence of both beneficial and detrimental mechanisms illustrates the complexity of microglia/macrophage phagocytosis in CNS disease. Other factors such as sex, aging, and disease progression may further complicate the function of phagocytes in brain injury.^18^, ^19^, ^20^ Thus, understanding microglia/macrophage‐associated phagocytosis after brain injury at the molecular and cellular levels may accelerate the discovery of better therapeutic strategies. In this review, we examine the available literature on microglial/macrophage phagocytosis in acute brain injuries, with a focus on the underlying mechanisms and therapeutic modalities that offer hope for clinical translation.

## 2 MICROGLIA/MACROPHAGE PHAGOCYTOSIS SIGNALING IN RESPONSE TO CNS PATHOLOGIES

### 2.1 Signals that activate or hinder phagocytosis: “Find‐me,” “eat‐me,” and “do not‐eat‐me” signals

**FIGURE 1:**
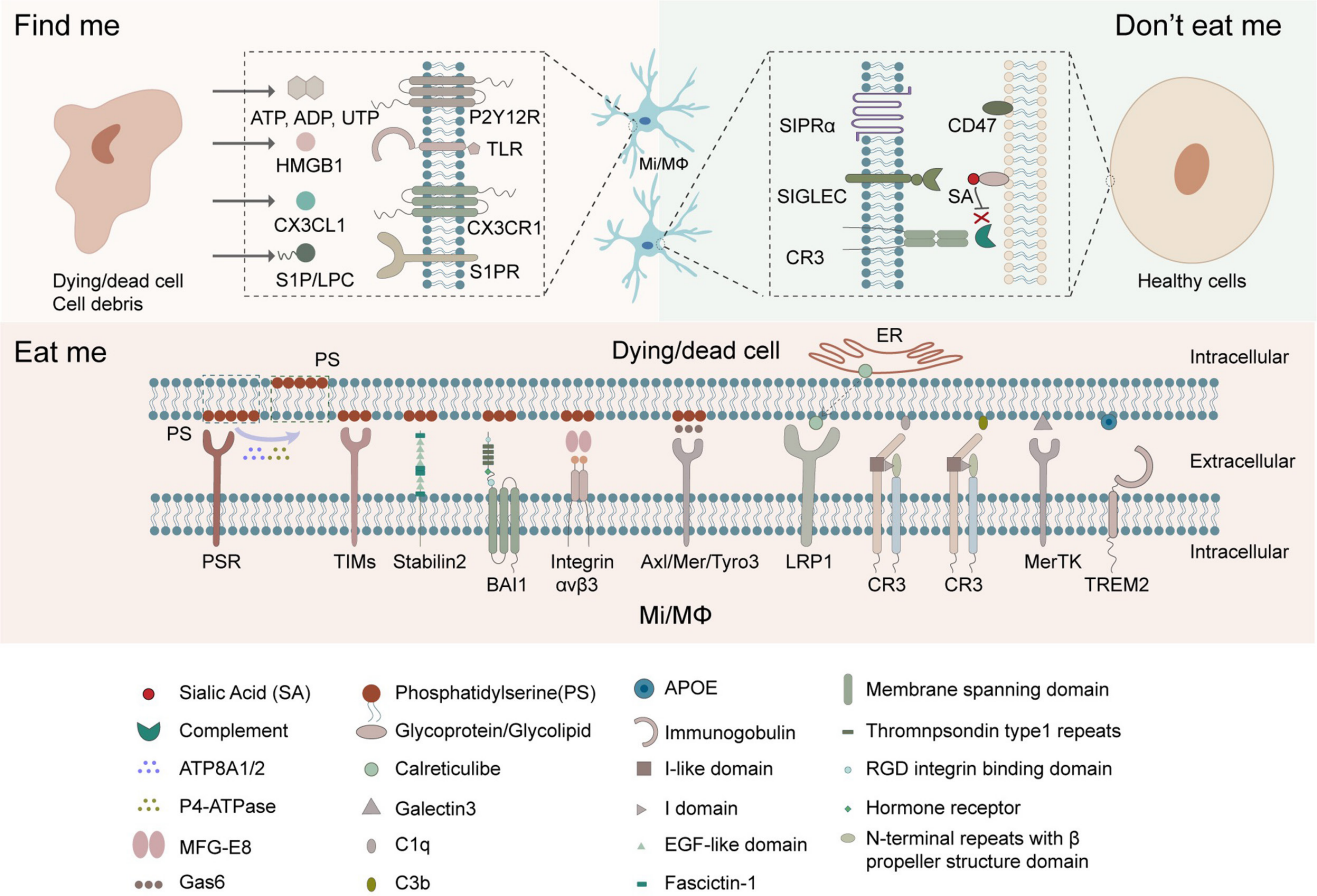
Find‐me, Eat‐me, and Do not‐eat‐me signals implicated in microglia/macrophage phagocytosis pathways after brain injury. Find‐me signals are instrumental in the recognition of chemotactic modulators, such as nucleotides, CX3CL1, and other molecular signals, including S1P/LPC and HMGB1 released by dying/dead brain cells. Find‐me signals are recognized by microglia/macrophage receptors P2Y12, TLR, CX3CR1, and S1PR, resulting in chemotaxis of microglia/macrophages to injured brain areas. Eat‐me signals are released or expressed by dead/dying brain cells (mostly neurons). When phosphatidylserine (PS) is flipped and exposed on the outer layer of the cell membrane, it is recognized by multiple microglia/macrophage receptors that initiate the phagocytosis process. Do not‐eat‐me signal pathways inhibit phagocytosis and involve sialylated glycoproteins and lipids. CD47 interacts with microglial receptor signal‐regulatory protein alpha (SIRPα) to inhibit phagocytosis

Dead or stressed neurons are known to release “find‐me” injury signals, such as nucleotides, sphingosine‐1‐phosphate (S1P), high mobility group box 1 (HMGB1), lysophosphatidylcholine, and CX3CL1,^3^ to recruit neighboring microglia and blood‐derived macrophages to the brain injury sites. Dead/dying brain cells or cell debris has specific “eat‐me” tags that attract microglia/macrophages and trigger an inflammatory response and the phagocytic process.^6^ However, viable neural cells express “do not‐eat‐me” signals to deter aberrant “eat‐me” processes and mitigate phagocytic injury^7^ (Figure 1), as discussed below.

#### 2.1.1 “Find‐me” signals

Damaged or stressed‐but‐viable neurons release nucleotides, such as adenosine triphosphate (ATP), adenosine diphosphate (ADP), or uridine triphosphate (UTP), as typical “find‐me” signals that guide microglia/macrophages to move or extend their processes toward the injury sites.^8^, ^21^ During development, neuronally released nucleotides participate in synaptic pruning by activating the P2Y12 receptor on microglia.^22^ However, in the context of ischemic brain injury, the P2Y2 receptor for nucleotides is upregulated on microglia.^3^ Indeed, neurons release nucleotides under both physiological and pathological conditions, and this response might be associated with microglial activation.

Chemokine CX3CL1 (fractalkine) is a strong chemoattractant “find‐me” signal. Both neurons and synapses release CX3CL1 to activate the microglial CX3CR1 receptor.^23^ In neurological disorders such as stroke and Alzheimer's disease,^23^, ^24^ downregulation of microglial CX3CR1 signaling decreases the activation of CNS resident microglia and the recruitment of peripheral macrophages, thereby improving neurofunctional outcomes in rodent animal models. However, the impact of CX3CR1 is complex, because CX3CR1 depletion impairs microglial phagocytosis and causes synaptic dysfunction in the adult hippocampus.^25^ Furthermore, during neurodevelopment, CX3CL1‐CX3CR1 interactions and microglial phagocytosis are essential in synapse pruning; hence, disabling this pathway leads to insufficient or delayed phagocytosis and autism‐like behavior in mice.^21^

Other “find‐me” signals such as sphingosine‐1‐phosphate (S1P) and lysophosphatidylcholine (LPC)^3^ are related to macrophage recruitment after stroke, and dimerized ribosomal protein S19, endothelial monocyte‐activating polypeptide II, or tyrosyl rRNA synthetase (TyrRS) serve as broad “find‐me” signals that mainly induce macrophage phagocytosis in the periphery.

#### 2.1.2 “Eat‐me” signals

Phosphatidylserine (PS) is a major phospholipid in the inner leaflet of the neuronal plasma membrane^26^, ^27^ and an essential “eat‐me” signal once flipped to the outer leaflet of endangered cells and exposed on their cell surface. Dead/dying cells or cell components actively display PS on the outer cell surface to induce microglia/macrophage phagocytosis.^7^, ^15^ PS can also reversibly translocate to the surface of cells by translocases, thereby exposing the signal to surrounding microglia/macrophages.^15^ Type 4P‐typease (P4‐ATPase), ATP8A1 and ATP8A2 are gatekeepers of the “eat‐me” signals and prevent their exposure on the cell surface to avoid triggering phagocytosis.^15^ Oxidative stress, DNA damage, intracellular calcium buildup, ATP depletion, and caspase‐induced apoptotic cell changes can all cause irreversible translocation of PS to the cell surface and induction of microglial/macrophage phagocytosis.^15^, ^28^, ^29^ Although reversible PS exposure can occur in response to nontoxic stimuli,^15^ the persistent presence of PS on stressed‐but‐viable neurons^28^ might be associated with delayed neuronal death and long‐term neurodegenerative consequences in stroke.^16^ Thus, blockade of the steps involved in PS exposure might rescue endangered neurons and prevent neurodegeneration.^30^

PS is recognized by engulfment receptors on phagocytes. Phagocytic PS receptors either directly recognize PS or interact with PS through bridging proteins.^31^ Several receptors such as the phosphatidylserine receptor (PSR),^32^ the T‐cell immunoglobulin and mucin (TIM) family members,^33^, ^34^ Stabilin2,^35^ and brain angiogenesis inhibitor I (BAI‐I),^36^ may directly recognize PS. Indirect PS recognition can also occur via bridging proteins such as milk fat globule‐EGF factor 8 (MFG‐E8) and growth arrest‐specific gene 6 (Gas6).^37^, ^38^ These bridging proteins have two binding domains that cross‐link PS with its receptors on phagocytes. For example, MFG‐E8 binds to PS on apoptotic neurons and engages integrin αvβ3 on microglia, resulting in microglial phagocytosis of apoptotic neurons. Gas6 bridges the PS on apoptotic cells to the Axl/Mer family of tyrosine kinases (Mer, Axl, and Tyro3) on microglia, thereby stimulating microglial clearance of apoptotic neurons.

It remains uncertain whether PS exposure alone is sufficient to induce phagocytosis. Some studies suggest that PS by itself can elicit phagocytic responses in macrophages; however, others show that not all cells with exposed PS are taken up by phagocytes.^39^, ^40^ Thus, a second signal may work synergistically with PS.^41^ Alternatively, PS may have to be modified to induce phagocytosis.^42^

Cell surface calreticulin is an alternative “eat‐me” signal and interacts with low‐density lipoprotein receptor‐related protein 1 (LRP1) on microglia/macrophages to initiate the phagocytic process.^15^, ^43^ The calreticulin/LRP phagocytic signaling pathway is highly associated with the elimination of stressed‐but‐viable neurons and long‐term neuronal loss in stroke.^21^, ^44^ In Aβ‐induced phagocytosis of live neurons in vitro, blocking calreticulin from the neuron surface or blocking LRP1 on microglia/macrophages successfully inhibited the death of live neurons by phagocytosis.^44^ In healthy brain cells, calreticulin is present predominantly in the endoplasmic reticulum, but it is translocated to the cell surface in response to endoplasmic reticulum stress, apoptosis, or pro‐inflammatory signaling.^15^, ^17^ Calreticulin also serves as an opsonin that interacts with galactose on the surface of tumor cells and facilitates macrophage phagocytosis.^45^ However, free calreticulin can also bind to microglia and block microglial phagocytosis of neurons,^44^ offering a potential therapeutic strategy when calreticulin/LRP is overactivated and massive neuronal death ensues.

Complement, galectin‐3, and apolipoprotein E are essential opsonins that enhance phagocytosis by binding cell components or cells that are destined for engulfment. The complement component C1q strengthens signal recognition by binding to cell surface PS, calreticulin, or a desialylated cell surface, and promotes microglial phagocytosis through the production of C3b.^21^, ^46^ C1q can also directly bind to the functional phagocytic receptor complement receptor 3 (CR3) on the microglial cell surface, and induce phagocytosis.^47^ In the CNS, complement proteins deposit on synapses and promote synaptic pruning or elimination during neurodevelopment or neurological disease.^48^, ^49^ The key step in complement‐opsonin activation is the conversion of C3 to C3a and C3b, where C3a recruits and activates microglia, whereas C3b opsonizes synapses and neurons to be captured by microglial CR3.^15^, ^21^, ^47^, ^50^

Galectin‐3 connects galactose residues found on the cell surface or cell debris with MER receptor tyrosine kinase (MerTK) on phagocytes.^15^ After ischemic brain injury, galectin‐3 is pivotal in mediating microglial activation and proliferation; galectin‐3 knockout significantly increases apoptotic neuron numbers by impairing microglial phagocytic ability.^51^ However, in traumatic brain injury (TBI), the release of galectin‐3 is associated with greater neuronal loss and worse neurological function.^52^

In human cells, apoliprotein E (ApoE) was recently found to interact with the microglial TREM2 receptor.^53^ Brain cells that bind to ApoE promote microglial migration and enhance phagocytosis by activating the TREM2 receptor on microglia.^53^, ^54^ In neurodevelopment, astrocytes express ApoE and regulate its phagocytic ability via interaction with ApoE, while participating in homeostatic synapse pruning, clearance, and turnover.^55^

##### Do not‐eat‐me signals

Neuronal transmembrane protein CD47 and sialic acid are recognized “do not‐eat‐me” signals that counterbalance “eat‐me” signals and temper phagocytosis‐induced neuronal injury.^15^ CD47 interacts with signal‐regulatory protein alpha (SIRPα) on microglia/macrophages and inhibits phagocytosis. Apart from efferocytosis of apoptotic cells, the CD47‐SIRPα axis was recently found to participate in negative regulation of local membrane excision by microglia during developmental synaptic pruning.^56^, ^57^ Synapses also express CD47 and escape microglial phagocytosis during development. Expression of CD47 on myelin debris may hinder its clearance and contribute to Wallerian degeneration.^58^ In hemorrhagic stroke, downregulation of CD47 expression boosts the number of phagocytic microglia/macrophages, enhances erythrocyte phagocytosis, and fosters the resolution of hematomas and other blood components.^59^, ^60^, ^61^, ^62^, ^63^ However, activation of CD47 may influence brain injury and recovery through other mechanisms. In ischemic stroke, the CD47 signaling pathway may contribute to neutrophil extravasation into the brain parenchyma and amplify neuroinflammation.^64^, ^65^ After traumatic brain injury, CD47 is thought to modulate trans‐endothelial migration of neutrophils and cerebrovascular remodeling at late injury stages.^64^, ^66^ The pleiotropic functions of CD47 in different brain cell types presents an opportunity to modulate spatially distinct aspects of inflammation by targeting a single molecule.

The cell surface of healthy neurons has sialic acid residues integrated into glycoproteins and glycolipids. Sialylation of the cell surface blocks phagocytosis by interacting with sialic acid‐binding immunoglobulin‐like lectins (SIGLECs) on the microglial cell surface^17^ and inhibiting the binding of certain opsonins, C1q, C3b, and galectins,^67^ whereas desialylated glycoprotein on neurons amplifies the “eat‐me” signal and promotes phagocytosis.

Other “do not‐eat‐me” signals include fractalkine, which is usually expressed by healthy brain cells. Conversely, stressed or damaged cells may lose fractalkine function and encourage inflammatory phagocytosis by microglia.^68^

### 2.2 Microglia/macrophage receptors in the phagocytosis pathway

Specific microglia/macrophage receptors are engaged in different phagocytic processes.^15^, ^69^ After brain injuries, removal of dead/dying or stressed‐but‐viable neurons requires the release of Milk Fat Globule Factor‐E8 (MFG‐E8) from microglia/macrophages. MFG‐E8 then recognizes phosphatidylserine “eat‐me” signals on neurons.^15^, ^70^ Both MerTK and MEF‐E8 are transiently upregulated following transient brain ischemia.^16^ Vitronectin receptors (VNR) are also upregulated on the microglial surface in brain disorders and bind phosphatidylserine on the neuronal surface.^70^

As microglia/macrophages engulf invading neutrophils or their debris, they are a major determinant of neutrophil accumulation after ischemic stroke. Microglial phagocytosis of invading neutrophils requires VNR and lectins.^5^ The physiological removal or remodeling of synapses and neurites during development or neuroplastic changes usually requires C1q, C3b and CR3, whereas clearance of neuronal precursors involves oxidant‐associated caspase activation, CR3 and DAP12.^15^, ^71^, ^72^ A comprehensive understanding of these distinct receptors and phagocytic pathways is especially important under conditions of brain injury.

#### 2.2.1 Toll‐like receptors (TLRs)

Toll‐like receptors (TLRs) belong to the family of pattern recognition receptors (PRRs), which recognize pathogen‐associated molecular patterns (PAMPs) to activate the innate immune response.^73^ Microglia/macrophage TLRs are also strong inflammatory receptors and induce production of pro‐inflammatory cytokines in the stroke brain.^74^ The observation that inflammatory receptors on microglia/macrophage initiate phagocytosis suggests that neuroinflammation and phagocytosis can interact and even join forces in various disease phases after injury.^15^, ^16^, ^30^ TLRs play a fundamental role in innate immune responses and are exclusively expressed on antigen presenting cells (APCs).^75^ Activation of TLR4 accelerates the clearance of myelin and premature engulfment of healthy neurons in Aβ‐induced phagocytosis.^76^ In neuroinflammation, stimulation of microglia with TLR ligands in vitro blunts their ability to distinguish between healthy and dead/dying neurons, thus promoting inappropriate cell phagocytosis.^15^, ^44^ TLR‐activated microglia may release oxidants that cause target cells to expose PS as an “eat‐me” signal and upregulation of MFG‐E8, potentially magnifying phagocytic injury.^7^, ^15^, ^77^ Given that microglial TLR affects inflammation and phagocytosis, a better understanding of the roles of TLR in the brain is warranted, as targeting or modulating its effects may be beneficial in models of neurodegenerative disease.^78^

#### 2.2.2 Scavenger receptors

Scavenger receptors (SRs) are involved in the uptake of negatively charged macromolecules and low‐density lipoprotein (LDL).^79^ Microglial SRs include macrophage receptor with collagenous structure (MARCO), SR‐B3 (CD36), and macrosialin (CD68). The activation of MARCO triggers reorganization of the microglial cell cytoskeleton, which is critical for phagocytosis.^80^ Increased expression of MARCO in mouse cortex following ischemic stroke is consistent with microglial clearance of debris and degenerating cells in the stroke brain.^81^ In addition, stimulation of CD36 signaling can promote microglial phagocytic capacity and is an important mechanism underlying blood clearance after intracerebral hemorrhage.^82^ Carbon monoxide, a potentially neuroprotective agent at low concentrations, was found to control microglial erythrophagocytosis by regulating cell surface expression of CD36 and also reduced the severity of hemorrhagic injury.^83^ Pro‐inflammatory cytokines such as TNF‐α downregulate microglial CD36 expression, leading to impairments in microglial engulfment of the hematoma following intracerebral hemorrhage.^84^

Oxidatively modified lipoproteins are neurotoxic and a rapid clearance of oxidized lipoproteins is therefore vital for proper CNS function.^85^ Oxidized lipoproteins in the periphery are effectively removed from the circulation by SR‐expressing liver cells; however, the mechanism underlying the clearance of modified lipoproteins in the stroke brain remains elusive.

In addition to the aforementioned receptors, other receptors may also regulate microglial phagocytosis. For example, CD22 (Siglec 2) is a negative regulator of phagocytosis and its expression is upregulated on aged microglia.^86^, ^87^ CD22 also mediates the antiphagocytic effect of α2,6‐linked sialic acid, and inhibition of CD22 promotes clearance of myelin debris, amyloid‐β oligomers, and α‐synuclein fibrils in vivo.^86^, ^88^ Long‐term CNS delivery of an antibody that blocks CD22 function reprograms microglia toward a homeostatic transcriptional state and improves cognitive function in aged mice.^86^

#### 2.2.3 Triggering receptor expressed on myeloid cells 2 (TREM2)

TREM2 is primarily expressed on myeloid cells and belongs to the lectin‐like immunoglobulin superfamily.^89^ TREM2 plays a critical role in myelin processing by binding lipids that are components of the myelin sheath (e.g., sulfatides and PS) and signaling through its co‐receptor DAP12 to activate a transcriptional profile.^90^, ^91^ TREM2 knockout suppresses microglia/macrophage numbers in ischemic brain injury and impairs phagocytic capacity by decreasing expression of CD68 and interactions with apoptotic cells.^89^, ^92^ TREM2 also regulates microglial cell activation in response to demyelination.^92^, ^93^, ^94^ TREM2 deficiency causes impaired clearance of myelin debris and axonal dysfunction in both young and aging mice.^89^ TREM2 deficiency also exacerbates ischemic damage and worsens neurological recovery in ischemic stroke models.^95^ In intracerebral hemorrhage, TREM2 activation attenuates neuroinflammation and neuronal apoptosis by activating PI3K/Akt.^96^ Taken together, microglial TREM2 may play a neuroprotective role in stroke and is a promising therapeutic target for the treatment of acute brain injuries.^97^, ^98^

#### 2.2.4 Purinergic receptors

Following brain damage, injured neurons release uridine diphosphate (UDP), which binds to and activates microglia/macrophage P2Y6 receptors and induces formation of the phagocytic cup.^15^, ^99^ This signaling pathway is important for debris clearance and functional recovery after ischemic stroke.^100^ Inhibition of the P2Y6/UDP complex decreases microglial phagocytosis and protects the brain against ischemic stroke and other CNS diseases,^16^, ^100^, ^101^, ^102^ perhaps by mitigating production of inflammatory cytokines such as IL‐1β, TNF‐α, and IL‐6.^100^ P2Y6R‐mediated phagocytosis may be a therapeutic target for ischemic stroke at the acute injury stage. However, further studies are warranted to investigate the role of P2Y6R in both the acute and chronic stages of stroke.

#### 2.2.5 Phosphatidylserine receptor

Asymmetric distribution of phospholipids across the plasma membrane is a unique characteristic of eukaryotic cells.^103^ As discussed above, phosphatidylserine is on the inner leaflet of the plasma membrane under physiological conditions, but it is flipped to the cell surface before phagocytosis under apoptotic conditions.^27^ The recognition of phosphatidylserine on apoptotic cells and the subsequent phagocytosis of apoptotic cells by microglia are mediated by a phosphatidylserine receptor (PSR).^104^ Microglial PSRs include TIM1, TIM4, BAI1, and TAM receptor tyrosine kinases (RTKs), Axl and Mer (encoded by *Mertk* gene).^105^ PSR activation enhances microglial phagocytosis and modulates microglial activation toward an antiinflammatory phenotype.^104^ MerTK or MFG‐E8, both of which mediate PS‐recognition, are upregulated by microglia after brain ischemia and enhance delayed neuronal death.^16^ Blocking this pathway promotes brain recovery by mitigating delayed neuronal loss and is associated with improved cognitive function.^7^, ^15^ The TAM system is required for microglial recognition, response, and phagocytosis of Aβ plaques; TAM‐mediated microglial phagocytosis promotes the formation of dense‐core plaques.^106^

Phagocytosis of hippocampal axons is a major contributor to dementia progression and appears to be associated with an increased expression of the PS receptor BAI1 on microglia and PS exposure on hippocampal axons.^107^ Post‐stroke neuroplastic remodeling rewires neuronal connections, which is critical for recovery.^108^ Phagocytic engulfment of PS‐exposing cells can suppress pro‐inflammatory signaling but also cause neuronal death.^16^ Thus, aberrant microglial phagocytosis of neuronal components can occur under various pathological conditions, and successful modulation of these processes may promote the repair of injured brain tissues.

### 2.3 Transcription factors that modulate microglia/macrophage phagocytosis

**FIGURE 2:**
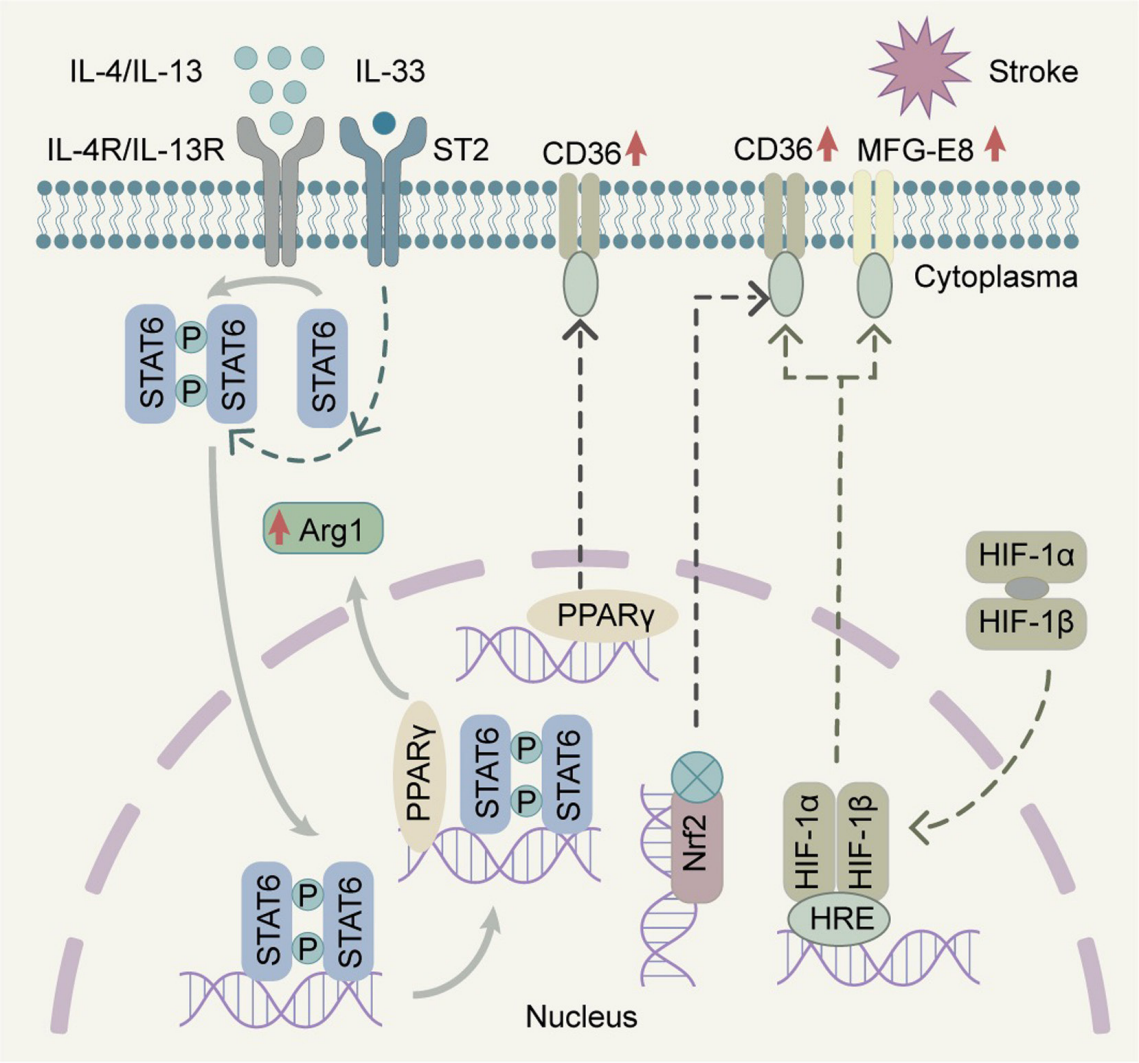
Microglia/macrophage transcription pathways that modulate phagocytosis following brain injuries. STAT6 signaling pathways might be modulated by IL‐4/IL‐13 or IL‐33, thereby modifying the phagocytic ability of microglia/macrophages. The STAT6 pathway also interacts with PPARγ and enhances microglia/macrophage phagocytosis in ischemic stroke. PPARγ, Nrf2, and HIF‐1α/β signaling induce expression of CD36 on the cell surface of microglia/macrophages, while HIF‐1α/β signaling may increase cell surface presentation of MFG‐E8 and magnify the phagocytic capability of microglia/macrophages following stroke

After “eat‐me” signals are recognized by phagocytic receptors on microglia/macrophages, actin polymerization is triggered and forms a phagocytic cup prior to the final phagocytotic process.^7^ Once there is closure of the phagocytic cup, the formed phagosome undergoes a process of maturation with endosomes and lysosomes before degrading the engulfed target.^109^ Regulation of this process may be another therapeutic target in brain disorders (Figure 2).

#### 2.3.1 Signal transducer and activator transcription 6 (STAT6)

Signal transducer and activator transcription 6 (STAT6) belongs to the Signal Transducer and Activator of Transcription family of proteins, which is principally activated by two cytokines, interleukin‐4 (IL‐4) and interleukin‐13 (IL‐13).^110^ STAT6 has multiple functions in lymphocytes such as T cells, B cells, and myeloid cells.^111^ STAT6 is also a key enhancer of erythrocyte engulfment after intracerebral hemorrhage, and the IL‐4/STAT6 axis promotes long‐term recovery in models of ICH.^112^ IL‐1 receptor‐like 1 activation is likely the key downstream step in IL‐4/STAT6‐conferred hematoma clearance. Accordingly, intranasal IL‐4 treatment or other STAT6 activators may be clinically feasible therapeutics for ICH.^112^ In ischemic stroke models, the STAT6/arginase1 (STAT6/Arg1) pathway is engaged in phagocytic clearance of dead/dying cells by both microglia and infiltrating macrophages, and upregulation of STAT6/Arg1 signaling ameliorates brain infarction and enhances long‐term neurofunctional recovery.^6^ The expression of STAT6 and the activity of its related signaling are increased after both ischemic and hemorrhagic strokes and are mechanistically linked to microglial/macrophage phagocytosis and superior neurofunctional outcomes. Treatment options targeting phagocytic STAT6 may therefore alleviate neurofunctional deficits associated with brain injuries. In particular, IL‐4‐enhanced microglia/macrophage phagocytosis may have good translational potential, given that IL‐4 has shown excellent safety profiles in multiple phase II clinical trials.^113^

#### 2.3.2 Peroxisome proliferator‐activated receptor‐γ (PPARγ)

Peroxisome proliferator‐activated receptor‐γ (PPARγ) has multiple functional roles in health and disease, such as regulating fatty acid synthesis, glucose metabolism, and adipocyte differentiation.^114^ PPARγ induces monocyte differentiation into distinct macrophages with more efficient phagocytic capacities and promotes the antiinflammatory response.^114^, ^115^ In neurological disorders, PPARγ enhances microglia/macrophage‐afforded elimination of Aβ and decreases oxidative stress in preclinical Alzheimer's disease.^116^ PPARγ activation increases phagocytic CD36 expression and promotes the clearance of blood deposits by microglia/macrophages, thereby improving neurofunctional recovery in animal models of ICH.^115^, ^117^ In animal models of ischemic stroke, PPARγ activation with rosiglitazone treatment increases phagocytic CD36 expression in microglia and enhances the clearance of invading neutrophils.^118^

#### 2.3.3 Nuclear factor‐erythroid 2 p45‐related factor 2

The transcription factor nuclear factor erythroid 2‐related factor (Nrf2) is a regulator of cytoprotective antioxidant and antiinflammatory signaling pathways.^119^ The Nrf2 agonist sulforaphane upregulates the scavenger receptor CD36 and promotes phagocytosis of red blood cells (RBCs) by microglia after ICH.^119^ As microglia are the primary phagocytic effector cells responsible for hematoma resolution after ICH, effective clearance of RBCs and RBC‐derived toxins represents a promising therapeutic option.^112^ Treatment with 2‐cyano‐3, 12‐dioxooleana‐1, 9‐dien‐28‐oic acid (CDDO)‐ethylamide (CDDO‐EA), a novel Nrf2 activator, can confer neuroprotection against ischemic injury by augmenting HO‐1 expression and microglial polarization toward the M2 phenotype in a murine stroke model.^120^

#### 2.3.4 Hypoxia‐inducible factor‐1α

Hypoxia‐inducible factor (HIF) is a heterodimer composed of oxygen‐sensitive α (HIF‐α) and oxygen‐insensitive β subunits. Under hypoxic conditions, instead of being hydroxylated, HIF‐α interacts with HIF‐1β in the cytoplasm and then translocates into the nucleus, where it binds to hypoxia‐responsive elements (HREs) of HIF‐targeting genes.^121^ HIF‐1α facilitates microglial phagocytosis by upregulating expression of CD36 and/or MFG‐E8, both of which contain HREs and promote stroke recovery during the acute stage.^122^ HIF‐1α also engages in the metabolic regulation of microglia.^123^ HIF increases glycolytic enzymes involved in energy metabolism and balances energy supply and demand.^124^ The mTOR‐HIF‐1α axis is also implicated in microglial metabolic reprogramming in AD, resulting in diminishment of microglial phagocytosis of Aβ.^125^ Further investigation of HIF‐1α is warranted to assess the beneficial aspects of microglial metabolic regulation by HIF‐1α in brain disorders.

## 3 MICROGLIA/MACROPHAGE PHAGOCYTOSIS IN DEFENSE AGAINST BRAIN INJURIES

### 3.1 Microglial phenotype after phagocytosis

Microglia play multifaceted roles in the pathogenesis of secondary brain injury at both acute and chronic stages.^126^ Together with recruited macrophages, microglia are the primary triggers of neuroinflammation in the CNS,^31^ which can be activated within minutes after stroke.^127^ Microglial proliferation reaches a peak at 48–72 h after stroke and can last for several weeks.^128^ Microglia are mainly detected in the area of ischemic core and then extend to the peri‐infarct region over time.^129^ Microglia assume distinct (sometimes polarized) phenotypes as an important warmup process before they join the battle, including transitioning between pro‐inflammatory and antiinflammatory profiles.^130^ Signaling pathways such as IFN‐γ and STAT1 promote a phenotype designated as “M1,” which is associated with secretion of pro‐inflammatory cytokines such as TNF‐α, IL‐1β, and IL‐12^131^, ^132^ to present antigens and kill pathogens.^133^ Conversely, IL‐4, IL‐10, or IL‐13 can mediate “M2” polarization and the secretion of neuroprotective factors, such as GDNF, BDNF, IL‐10, and TGF‐β^134^, ^135^ to facilitate tissue regeneration after injury.^136^ Both M1 and M2 microglia/macrophages express phagocytic receptors, but the M2 phenotype is associated with better phagocytic abilities and dead neuron clearance than the M1 phenotype.^137^ Phagocytosis mediated by the M1 phenotype may encourage inappropriate phagocytosis of viable neurons,^15^ while phagocytosis mediated by the M2 phenotype may be neuroprotective via antioxidative functions.

### 3.2 Does phagocytosis promote or hinder CNS function?

In the acute and subacute stages following brain injury, microglia/macrophages may exacerbate tissue damage by releasing pro‐inflammatory cytokines, and yet, these versatile cells also assist in tissue repair and vascular remodeling.^138^ Bulk RNA sequencing data suggest that macrophages in the mouse brain are subject to reprogramming after experimental stroke and transform into a phenotype with enhanced efferocytotic activity.^3^, ^139^ Resident microglia are responsible for more phagocytic activity after stroke compared with hematogenous macrophages, but both cell types can contribute to debris clearance.^140^

One important phagocytic function for microglia is the engulfment of apoptotic cells and debris from broken myelin, which prevents the release of cytotoxic intracellular contents.^141^ Similarly, microglia eliminate neurotoxic blood products by phagocytosing and processing extravasated erythrocytes in ICH, preventing their lysis and the ensuing neurotoxicity.^142^ The infiltration of other myeloid cells such as neutrophils contributes to pro‐inflammatory neuronal damage at the acute phase of stroke.^143^ Microglia also have the power to remove infiltrating neutrophils via phagocytosis, thereby mitigating neutrophil‐mediated neurovascular destruction after brain injury.^10^, ^144^ Notably, neutrophils can escape from microglial engulfment via plasminogen activator inhibitor type 1 (PAI1)‐dependent downregulation of vitronectin receptor (VNR) after brain injury.^145^ On the contrary, microglia also directly engulf endothelial cells and may facilitate disintegration of blood vessels and breakdown of the blood brain barrier (BBB), which would accelerate further infiltration of circulating immune cells into brain parenchyma.^146^ After ischemia, neurons expose PS on their outer plasma membrane via the calcium‐activated phosphatidylserine scramblase TMEM16F. This process may allow microglia to phagocytose stressed (but still viable) neurons in the penumbra and exacerbate functional deficits after ischemia and reperfusion.^28^ On the contrary, the impact of microglial phagocytotic actions toward live cells upon long‐term stroke outcomes remains unknown, and the clearance of stressed‐but‐viable cells may or may not facilitate long‐term rewiring.

In the chronic stages of brain injury, microglia/macrophages have a multipronged impact on neurogenesis, angiogenesis, and neuroplasticity in brain tissues.^147^, ^148^ Microglia may modulate neuronal and synaptic functions after stroke by stimulating the proliferation of neural progenitor cells (NPCs).^149^ Indeed, neurogenesis is accelerated after brain damage.^150^ Microglia in the ipsilateral subventricular zone (SVZ) promote neurogenesis through upregulation of TGF‐α,^151^ while those in the peri‐infarct areas exert the opposite effects. In the ischemic penumbra, perivascular microglia promote blood vessel disintegration via upregulation of CD68 expression^146^ but in the contralateral nonischemic hemisphere, microglia release enhance angiogenesis via VEGF.^152^ Some of the synapses within the ischemic areas display enhanced turnover rates after contacting microglia, suggesting that microglia are involved in synaptic pruning after stroke.^153^

Excessive phagocytosis of myelin sheaths can accelerate demyelination with detrimental consequences. Microglia selectively phagocytose myelin sheaths to sculpt myelination in homeostatic conditions,^154^ but they can also cause myelin damage by excessively engulfing myelin sheaths 14 days after stroke.^14^ On the contrary, when microglia/macrophages internalize cholesterol‐rich myelin debris, they can then synthesize sterols to reinforce remyelination by oligodendrocytes in multiple sclerosis (MS).^155^ Whether or not similar mechanisms exist in post‐stroke remodeling remains to be investigated.

In summary, microglia/macrophages play dynamic roles in brain injuries. They engulf dead/dying neurons and neuronal debris to reduce inflammation but may also phagocytose injured yet salvageable neurons in the ischemic penumbra. Although both brain resident microglia and infiltrating macrophages are involved in phagocytic activities following acute brain injury, most studies do not distinguish these two cell populations. However, microglia and macrophages may exhibit distinctive characteristics after brain damage.^112^ A thorough understanding of the molecular and cellular mechanisms underlying the regulation of microglia/macrophage phagocytosis after brain injury and the causal link between the temporospatial characteristics of phagocytosis and disease progression/recovery is warranted. The latter information may be leveraged in novel therapeutic strategies to promote the beneficial effects of phagocytosis while avoiding its detrimental effects.

## 4 MICROGLIA/MACROPHAGES TOIL IN SUPPORT OF BRAIN REPAIR

The brain is a lipid‐rich organ. A large proportion of lipid is found within the myelin sheath.^156^ Lipid uptake or phagocytosis by microglia/macrophages clears the lipidic myelin debris after brain injury. Subsequent lipid metabolism by microglia/macrophage supports axon regeneration, neurogenesis, and rewiring of the neuronal network. Microglia directly interact with synapses during development and under disease conditions. Furthermore, they help to clear disorganized synapse structures, enhance synaptic pruning, and create a favorable extracellular matrix environment for restoration of the anatomical structure and physiological function of the brain after injury.^157^

### 4.1 Fat processing after myelin phagocytosis

Persistent demyelination following brain injury impairs neurofunctional recovery. A successful remyelination process following brain injury involves the clearance of myelin debris, the recycling of lipids by microglia and macrophages, and the regeneration of oligodendrocytes, all of which rebuilds the myelin sheath and rewires functional neuronal circuits.^155^, ^156^ A recent single‐cell RNA sequencing study suggests that microglia/macrophages upregulate genes coding for ApoE and Lpl, as well as other phagocytic signature genes associated with lipid clearance and metabolism and extracellular matrix reshaping.^156^ Because the CNS cannot use lipids from non‐brain sources, microglia/macrophage‐afforded recycling of lipid debris from broken myelin is of great importance in neuroplastic recovery after brain injury. A timely activation of the pro‐inflammatory program in microglia/macrophages appears to be required for myelin clearance, oligodendrogenesis, and remyelination.^158^ Microglia/macrophages that actively participate in myelin repair (myelin clearance and remyelination) exhibit increased lipid droplet accumulation and pro‐inflammatory characteristics, including increased TREM2 expression.^93^, ^159^ Microglia/macrophages appear to facilitate myelin repair through sequential actions involving the engulfment of myelin debris and synthesis and production of desmosterol, the precursor of cholesterol.^155^ Thus, pharmacological manipulations that enhance myelin repair by regulating lipid metabolism in microglia/macrophages may have clinical potential against brain injury.

### 4.2 Synaptic pruning and remodeling

Synaptic pruning and remodeling are pivotal in neuroplastic recovery after injury. During neural development, microglia help shape the neural network by phagocytosing excess synapses, dendrites, and axons, and refining the neural circuitry. Microglia also regulate myelination during developmental myelin sheath formation, contributing to neural circuit refinement and adaptation.^160^ Synaptic pruning and remodeling are activated during the recovery phase following brain injuries. The successful rewiring of surviving neural networks and the recruitment of functional synapses after injury are positively correlated with neurofunctional recovery.^108^ Several mechanisms are believed to underlie regulation of synaptic pruning and remodeling by microglia/macrophages, such as the complement opsonin C3 and CR3.^17^ Finally, microglia support synaptogenesis and pruning after brain injury by regulating the extracellular matrix. Upon activation by the cytokine IL‐33 following brain injury, microglia upregulate expression of extracellular matrix proteases that promote turnover of pathological extracellular matrix proteins to create a more favorable microenvironment for synapse plasticity and neuronal network rewiring.^161^

## 5 SUMMARY

In this review, we have summarized the molecular mechanisms that are engaged during microglia/macrophage phagocytosis after brain injury and their potential roles in functional outcomes. We have also described phagocytic pathways that play both detrimental and beneficial roles in brain injury and recovery. However, there are important knowledge gaps in our understanding of the role of microglia/macrophage phagocytosis in acute brain injury. First, most studies on microglia/macrophage phagocytosis do not distinguish between microglia and macrophages. CNS resident microglia and infiltrating bone marrow‐derived macrophages may display distinctive phagocytotic profiles under different brain injury conditions. For example, infiltrating bone marrow‐derived macrophages appear to dominate over microglia in the phagocytosis of red blood cells and hematoma resolution after ICH.^112^, ^162^ A similar trend has been reported in animal models of ischemic stroke,^163^ where resident microglia might themselves be susceptible to an ischemic insult, thus impairing their phagocytic capabilities, while peripheral macrophages could be fully activated by damage signals from the brain. A better understanding of the diverse roles of CNS resident microglia and peripherally originating macrophages in phagocytosis may have important implications in fine‐tuning novel therapeutic strategies against brain injuries.

Second, border‐associated macrophages (BAMs) residing in the dura mater, subdural meninges, and choroid plexus represent another myeloid cell population with unique transcriptomes, cellular compositions, and phagocytic abilities compared with microglia and bone marrow‐derived macrophages.^3^, ^164^ Following stroke, both BAMs and bone marrow‐derived macrophages are recruited to the brain lesion site. Post‐stroke BAMs are more similar to microglia than bone‐derived macrophages in multiple aspects, including gene expression.^165^ However, in AD, a downregulation of phagocytosis‐related genes is observed in BAMs compared with microglia.^164^ Furthermore, a non‐negligible proportion of BAMs can be replaced by infiltrating monocyte/macrophages during brain injury, after BAMs have infiltrated the ischemic brain parenchyma.^166^

Third, most studies on ischemic or hemorrhagic stroke or on traumatic brain injury have used rodent models. However, there exist substantial differences in rodent versus human microglial biology. A recent single‐cell RNA sequencing study shows that non‐diseased human brain microglia express elevated levels of CCL2, CCL4, EGR2, and EGR3, suggesting that human microglia may have a chronic pro‐inflammatory baseline compared with rodent microglia.^167^ TGF‐β1 is an important cytokine expressed in mouse microglia but less is known about its function in human microglia.^168^ Furthermore, mouse microglia are robustly activated via TLR4 upon lipopolysaccharide stimulation, whereas human brain microglia are less responsive to TLR4 ligand binding.^167^

Fourth, aging and sex differences add on extra layers of complexity to microglia/macrophage phagocytosis in brain injuries, although these topics are under active investigation.^139^, ^169^ In aged individuals, the expression of phagocytosis receptors is altered,^170^ and phagocytic clearance of cell debris by microglia is impaired due to an overload of misfolded proteins, inadequate responses to stimuli such as systemic inflammation, and microglial overactivation in secondary brain injury.^20^, ^171^, ^172^ Sex differences are also a major but understudied factor impacting brain function and disease profiles. Male and female rodents are known to display distinct microglia distributions, motility, and functional activities at baseline and under disease conditions.^20^ Thus, further investigations of the impact of aging and sex differences on microglia/macrophage phagocytosis are warranted.

Fifth, the majority of stroke patients have at least one comorbidity, most of which are also independent risk factors for stroke.^173^, ^174^ Aging is a robust disease modifier that worsens stroke recovery.^115^ Aside from age‐related pathologies, almost all types of comorbidities, including hypertension and diabetes, are associated with chronic inflammation, microvascular dysfunction, metabolic disorders, and microglia/macrophage deregulation.^25^, ^77^, ^158^, ^175^ Mounting evidence demonstrates that aging and stroke comorbidities exert a significant impact on the phagocytic actions of microglia and macrophages. For example, diabetic conditions and resulting cerebral microbleeds encourage the infiltration and aggregation of phagocytic, galectin 3‐expressing macrophages from blood, to assist in the clearance of dysfunctional micro‐vessels.^176^ Type 2 diabetes is also a potent trigger of enhanced microglia/macrophage proliferation and shifts microglia/macrophages toward pro‐inflammatory phenotypes associated with unfavorable long‐term neurofunctional outcomes.^177^, ^178^ In addition, macrophages are key arbitrators in the pathophysiology of hypertension.^179^ Macrophages display abnormal activation and phenotype switching in hypertension; thus, further study is warranted on the phagocytic activities of macrophages in brain injury under conditions of preexisting hypertension.^180^, ^181^, ^182^

In conclusion, a better mechanistic understanding of phagocytosis may help us selectively target and manipulate microglia/macrophage function and hasten the safe and effective treatment of brain disorders in the clinic.

## CONFLICT OF INTEREST

None.

6

## Data Availability

Data can be obtained via corresponding author.
